# Chaos-assisted two-octave-spanning microcombs

**DOI:** 10.1038/s41467-020-15914-5

**Published:** 2020-05-11

**Authors:** Hao-Jing Chen, Qing-Xin Ji, Heming Wang, Qi-Fan Yang, Qi-Tao Cao, Qihuang Gong, Xu Yi, Yun-Feng Xiao

**Affiliations:** 1 State Key Laboratory for Mesoscopic Physics and Frontiers Science Center for Nano-optoelectronics, School of Physics, Peking University, 100871 Beijing, China; 2 Department of Electrical and Computer Engineering, University of Virginia, Charlottesville, VA 22904 USA; 3 T. J. Watson Laboratory of Applied Physics, California Institute of Technology, Pasadena, CA 91125 USA; 4 Collaborative Innovation Center of Quantum Matter, 100871 Beijing, China; 5 Collaborative Innovation Center of Extreme Optics, Shanxi University, 030006 Taiyuan, China; 6 Peking University Yangtze Delta Institute of Optoelectronics, Nantong, Jiangsu 226010 China; 7 Department of Physics, University of Virginia, Charlottesville, VA 22904 USA

**Keywords:** Microresonators, Solitons, Frequency combs, Nonlinear optics

## Abstract

Since its invention, optical frequency comb has revolutionized a broad range of subjects from metrology to spectroscopy. The recent development of microresonator-based frequency combs (microcombs) provides a unique pathway to create frequency comb systems on a chip. Indeed, microcomb-based spectroscopy, ranging, optical synthesizer, telecommunications and astronomical calibrations have been reported recently. Critical to many of the integrated comb systems is the broad coverage of comb spectra. Here, microcombs of more than two-octave span (450 nm to 2,008 nm) is demonstrated through *χ*^(2)^ and *χ*^(3)^ nonlinearities in a deformed silica microcavity. The deformation lifts the circular symmetry and creates chaotic tunneling channels that enable broadband collection of intracavity emission with a single waveguide. Our demonstration introduces a new degree of freedom, cavity deformation, to the microcomb studies, and our microcomb spectral range is useful for applications in optical clock, astronomical calibration and biological imaging.

## Abstract (web summary)

Here, the authors demonstrate the use of chaos to obtain 2-octave comb generation. The deformation lifts the circular symmetry and creates chaotic tunneling channels that enable broadband collection of intracavity emission with a single waveguide, introducing a new degree of freedom to microcomb studies.

## Introduction

Microresonator-based frequency comb (microcomb) uses Kerr nonlinearity to create parametric gain and offset cavity loss^1^. It has been demonstrated in various material platforms including silica^2^, CaF_2_ (ref. ^3^), MgF_2_ (ref. ^4^), high-index silica^5^, silicon nitride^6^, AlN^7^, diamond^8^, and LiNbO_3_^9^. The recent development of dissipated Kerr soliton microcombs^10–17^ has enabled applications ranging from spectroscopy to astronomy calibration^18–25^. The microcomb also provides a novel platform for nonlinear physics studies and has led to observations of Stokes solitons^26^, soliton crystals^27^, and soliton interactions^28^. In these demonstrations, the microcombs are generated in either circular-symmetric whispering-gallery-mode (WGM) cavities or waveguide-mode cavities, and are coupled out by phase-matched, evanescent couplers. The efficiency of the coupler drops significantly at wavelengths far from that of the pump laser. The same challenge is also imposed on microcavity harmonic generation, but it is often overcome by adding additional coupler^29^. However, this resolution does not directly applied to microcombs with broad spectral coverage. Complicated coupler has recently been designed and tested for octave-spanning microcombs^30,31^.

Asymmetric (deformed) cavity, first proposed by Nöckel and Stone^32^, can output emissions from whispering-gallery modes to free space without using evanescent couplers. Its applications in directional laser^33^, suppression of laser spatiotemporal instabilities^34^, light storage^35^, as well as single nanoparticle biosensing^36^ have been demonstrated. Generally, ray dynamics in a billiard together with the dynamic tunneling in the momentum space provides a versatile tool to understand the interesting phenomena in asymmetric microcavities^37,38^. Recently, it was revealed that the chaotic channels in the deformed cavity can assist broadband momentum transformation^39^, opening up new possibilities to resolve the challenge in outputting broadband nonlinear emissions from microcavities.

**Fig. 1:**
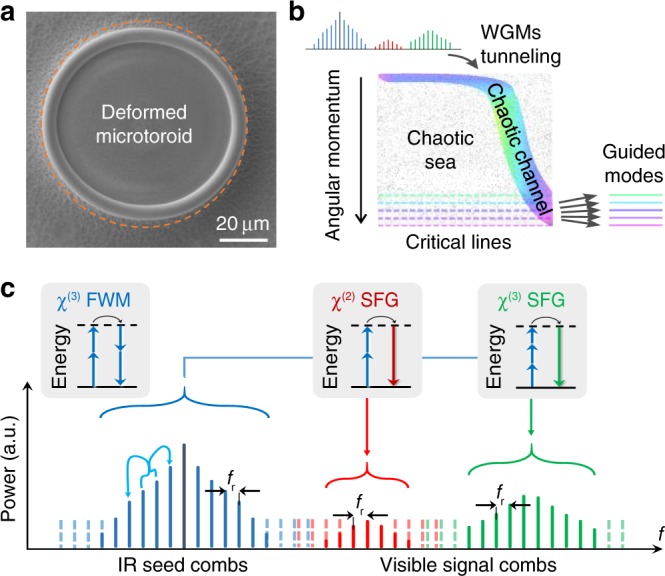
Schematic for broadband microcombs in the deformed microcavity. **a** Scanning electron microscope image of a deformed microresonator. As a comparison, a circular contour is shown in dashed orange line. **b** Conceptual illustration of chaos-assisted momentum transformation and dynamical tunneling from whispering-gallery-modes (WGMs) to chaotic states. **c** Schematic of the nonlinear frequency conversion from infrared (IR) to visible wavelengths in the deformed cavity with four-wave mixing (FWM) and sum-frequency generation (SFG, including harmonic generations as a special case).

In this Article, we report a microcomb spanning from 450 to 2008 nm in a deformed cavity (Fig. 1a). The microcomb is pumped near 1550 nm, and its frequency range is extended by nonlinearities^40–44^ of intracavity *χ*^(3)^ and symmetry-breaking induced *χ*^(2)^ (ref. ^45^) (Fig. 1c). Importantly, to output the broadband emission, the microcavity is deformed slightly to create a chaotic tunneling channel. Because of the chaotic motion, the angular momentum of light is not conserved, and it changes with time, covering a broad range of values (Fig. 1b). Once the angular momenta of the comb reach the corresponding critical lines of total internal reflections, the comb can be effectively collected by a nanofiber through nearly wavelength-independent refraction process^39^.

## Results

### Two-octave-spanning microcomb

The silica microtoroid resonator^46^ used in this experiment has principal (minor) diameter of 75 μm (4.2 μm) and its *Q*-factor is 1.6 × 10^7^ at 1550 nm. The microcavity is weakly deformed to create chaotic channels and the minimum diameter is 5.6% smaller than the maximum diameter. The details of the deformed cavity boundary shape are included in the “Methods” section. A tunable laser at 1550 nm is used as the pump. Its power is amplified to 1.6 W and is coupled into the cavity through a 720-nm-diameter nanofiber.

**Fig. 2:**
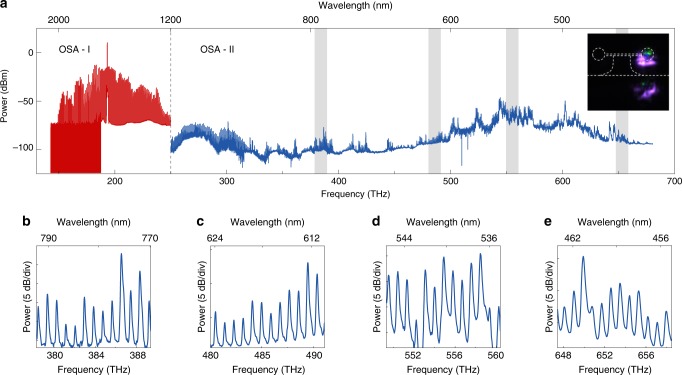
Optical spectrum of two-octave-spanning microcombs. **a** Complete optical spectrum of microcombs. Spectrum measured with Yokogawa optical spectrum analyzer (OSA) above 1200 nm is presented in red, while the shorter wavelength part is measured by an Andor spectrograph (shown in blue). The absolute power of the spectrum are calibrated at 1550 and 635  nm for the OSA and spectrograph, respectively. The power mismatch of the red and blue spectrum at 1200  nm is caused by the spectrograph efficiency variation over the broad spectral range. Inset: an image taken by a CCD camera on the side of the cavity. Green and violet emissions are apparent. **b–e** Zoom-in spectra of the gray areas are shown in panel **a**. The frequency ranges for **b**–**e** are 378.3–389.4, 480–491.1, 549.3–560.4, and 647.4–658.5 THz.

The optical spectrum of the microcomb is presented in Fig. 2a. The frequency span of the microcomb ranges from 150 to 670 THz. An optical spectrum analyzer (OSA, Yokogawa) is used to measure the spectrum above 1200 nm (shown in red), while the shorter wavelength part (shown in blue) is measured by a spectrograph (Andor). The power of the OSAs are calibrated at 1550 and 635 nm. The selection of the calibration wavelength is constrained by the collection of laser sources in our lab. The power mismatch at 1200 nm is likely to come from the variation of OSA’s efficiency versus wavelength. The noise floor of the spectrograph is also well characterized. The violet and green emission from the microtoroid can be observed directly by a CCD camera set on the side of the microtoroid (Fig. 2a, inset). The zoom-in spectra of the microcombs at 378.3–389.4, 480–491.1, 549.3–560.4, and 647.4–658.5 THz are shown in Fig. 2b–e.

**Fig. 3:**
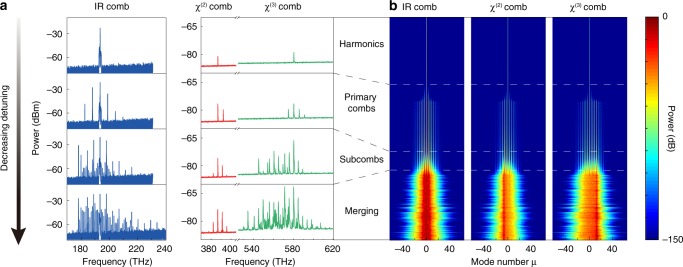
Formation process of microcombs. **a** Four representative optical spectra of microcombs. The amplitude of cavity-laser detuning is gradually decreased from top to bottom. The second and third harmonic generation is observed first, followed by the primary combs in all three frequency bands. Subcombs are then generated and they slowly merge with the decrease of detuning. **b** Simulated microcomb formation process. Pump detuning is scanned from shorter to longer wavelength, and the spectrum evolution of microcomb is presented. The intracavity power for three bands is normalized to the largest value of their own bands.

The formation process of broadband microcomb in deformed cavity is investigated both experimentally and numerically (Fig. 3). In this measurement, the frequency detuning of laser cavity is slowly decreasing while the optical spectrum is recorded. Four typical phases of microcomb are presented in Fig. 3. First, the thresholdless harmonic generation is observed at 386.9 and 580.5 THz as the frequency of pump light is 193.4 THz. With the decrease of detuning, the intracavity pump power reaches the parametric threshold and the primary comb lines are generated near the pump wavelength as well as the harmonic wavelengths. We refer the comb lines near the pump wavelength as seed comb, and the rest of the comb as signal comb. The signal comb is generated from sum-frequency generation (SFG) of the seed comb, and the four-wave mixing between the seed comb and the harmonics of the pump laser. Subcombs with comb spacing identical to cavity FSR appear in both seed comb and signal comb when further decreasing the detuning. The subcombs will eventually merge together in all three bands. The formation process of broadband microcomb is well reproduced in our numerical simulation of coupled Lugiato–Lefever (LL) equations (see Methods) and is presented in Fig. 3b. Note that the numerical simulation of intracavity light field is performed by employing a circular cavity without the effects of chaotic propagation. This configuration is an approximation for the weakly deformed high-*Q* cavity to reduce computational complexity.

**Fig. 4:**
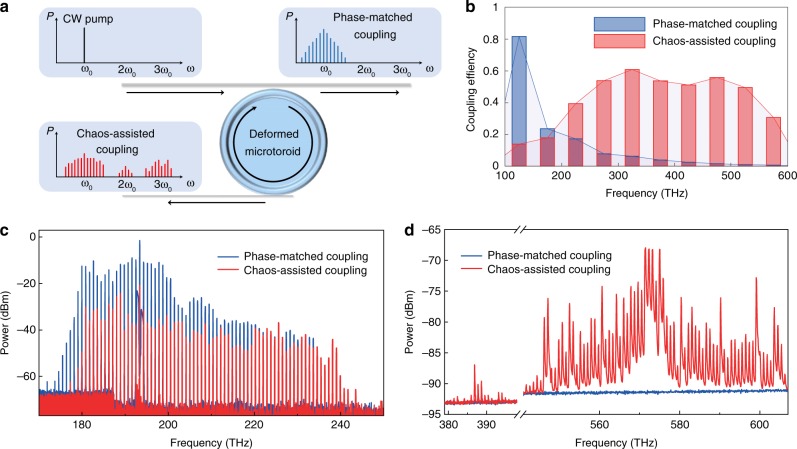
Comparison between chaos-assisted coupling combs and phase-matched coupling combs. **a** Conceptual schematic: a phase-matched microfiber is used to couple pump laser into the deformed cavity and it also collects comb emissions at the through port. A nanofiber is used at the drop port to output comb light through chaos-assisted coupling. **b** Three-dimensional finite difference time-domain (3D FDTD) simulation of the coupling efficiencies of both phase-matched coupling and chaos-assisted coupling. **c**, **d** Optical spectra of the frequency combs with phase-matched coupling (blue) and chaos-assisted coupling (red) in infrared (**c**) and visible (**d**) regions.

A comparison of comb output between phase-matched coupling and chaos-assisted coupling is demonstrated. In this experiment, a microfiber and a nanofiber are simultaneously coupled to the deformed cavity (Fig. 4a). The diameter of the microfiber is set to 1.5 μm to achieve phase-matched coupling with the whispering-gallery mode at 1550 nm. The 0.5-μm-diameter nanofiber has effective refractive index close to 1, and it is used to collect the comb light through chaos-assisted coupling. The coupling efficiency can be simulated by 3D FDTD method, and the result is presented in Fig. 4b. The efficiency for phase-matched coupling decreases rapidly with the increase of optical frequency, while the efficiency for chaos-assisted coupling maintains very well from near-IR to visible wavelengths. The dimension of the cavity is scaled down in this simulation and the detailed is described in the Methods. In the experiment, 300 mW pump power is sent into the cavity through the microfiber. The optical spectra are measured at both the through port and drop port and are shown in Fig. 4c, d. No comb signal in near-visible or visible band can be seen for the phase-matched coupling output. In comparison, intense comb lines are detected with chaos-assisted coupling.

### Observation of soliton formation steps

**Fig. 5:**
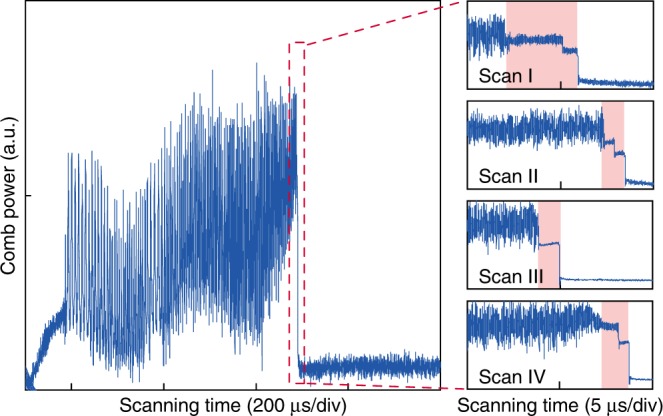
Observation of soliton formation steps. Experimental traces of the comb power are recorded when the pump laser frequency is scanned from blue to red detuning at a scanning speed of  ~10 nm/s. The right panels show the zoom-in traces of four representative soliton formation steps in different scans, of which the number and the amplitude of steps change from scan to scan.

Soliton formation in microresonators is critical to many microcomb applications for its high coherence and predictable spectrum envelope^16^. Here, we show that soliton formation could be feasible in a deformed cavity for the first time. In the experiment, the laser wavelength is scanned from blue-detuned to red-detuned regime, while the comb power is recorded on the oscilloscope. When the laser scanning rate is increased to  ~10 nm/s, the distinct soliton formation steps are observed. Upon entering the soliton regime, the comb power shows a “step” feature and the noise on the comb power disappears. Both single and multiple steps are observed and presented in Fig. 5. This points to evidence of dissipated Kerr cavity solitons in a microresonator^10^. It shall be noted that the transition from noisy comb to soliton regime is often complicated by thermal effect. The abrupt change in comb power leads to a rapid decrease of cavity temperature and thus a blueshift of the resonator frequency. As a result, the cavity-laser detuning quickly exits the soliton existence range and thus the soliton steps in our experiment are only  ~1 μs in duration. Several methods exist to overcome this thermal complexity, including pump power kicking^47^, rapid frequency scanning by using single-sideband modulator^48^, and auxiliary laser for thermal compensation^49,50^. Further investigation using these methods is needed in the future to stably access the soliton state in our cavity and to verify soliton mode-locking.

## Discussion

In summary, we have demonstrated microcomb with two-octave span ranging from 450 to 2008  nm in a deformed cavity. The microcomb wavelength range is useful for optical clock, medical imaging, and calibrating radial velocity for exoplanet detection. The deformation of cavity lifts the circular symmetry and introduces a new degree of freedom to the microcomb systems. This could be used as an additional knob for broadband dispersion engineering of microresonator. Preliminary indication of soliton formation is also shown in the deformed cavity. The combination of temporal solitons and chaos in deformed cavity in the future may provide new possibilities for optical soliton physics studies and microcomb applications.

## Methods

### Deformed cavity design

In a deformed cavity, the angle of reflection and the angle of following incidence are different when a light beam is reflected on the boundary of the cavity. As a result, the incident angle *χ* of reflection is not conserved, nor is the angular momentum quantity $\sin \chi$. This varying *χ* makes the intracavity ray dynamics highly sensitive to initial conditions, and the chaotic channel forms accompanied by rapid and broadband momentum transformation.

By using this momentum transformation, the effective mode index of light can be reduced from 1.44 (close to that of the WGMs) to nearly 1 (close to that of the nanofiber) in the chaotic channel. As a result, the light can be easily coupled into the nanofiber from the chaotic channel through the wavelength-independent refraction. This chaos-assisted broadband momentum transformation is provided in the cavity with its boundary designed as

1 $$R(\phi )=\left\{\begin{array}{l}{R}_{0}+{R}_{0}\sum _{i=2,3}{a}_{i}{\cos}^{i}\phi \,\,{\cos\phi \ge} 0\\ {R}_{0}+{R}_{0}\sum _{i=2,3}{b}_{i}{\cos}^{i}\phi \,\,{\cos}\phi <0\end{array}\right.,$$

where *R*_0_ is the size parameter, and *a*(*b*)_2,3_ are optimized as *a*_2_ = −0.1329, *a*_3_ = 0.0948, *b*_2_ = −0.0642, and *b*_3_ = −0.0224.

### Coupled LL equations in the presence of *χ*^(2)^ and *χ*^(3)^ nonlinear processes

The comb formation process is simulated with three normalized coupled LL equations, which include multiple *χ*^(2)^ and *χ*^(3)^ nonlinear effects, e.g. SFG, different frequency generation, self-phase modulation, and cross-phase modulation. In the equations, *μ*_*ab*_ and *μ*_*ac*_ describe coupling strength for *χ*^(2)^ and *χ*^(3)^ SFG; *μ*_*ba*_ and *μ*_*ca*_ for the reverse process; *ζ*_*ab*_ and *ζ*_*ac*_ denote cross-phase modulation for *χ*^(2)^ and *χ*^(3)^ combs, and the inverse process is described by *ζ*_*ba*_ and *ζ*_*ca*_. Self-phase modulation of *χ*^(2)^ and *χ*^(3)^ combs is described by *ζ*_*b*_ and *ζ*_*c*_. The coupled LLEs are as follows:

2 $$\frac{\partial }{\partial \tau }{\Phi }_{{{a}}}=(-1-i{\alpha }_{{{a}}}+i{\beta }_{{{a}}}\frac{{\partial }^{2}}{\partial {\phi }^{2}}+i{\left|{\Phi }_{{{a}}}\right|}^{2}+i{\zeta }_{{{ba}}}{\left|{\Phi }_{{{b}}}\right|}^{2}+i{\zeta }_{{{ca}}}{\left|{\Phi }_{{{c}}}\right|}^{2}){\Phi }_{{{a}}}+{f}_{0}+i{\mu }_{{{ba}}}{\Phi }_{{{a}}}^{* }{\Phi }_{{{b}}}+i{\mu }_{{{ca}}}{\Phi }_{{{a}}}^{* 2}{\Phi }_{{{c}}},$$

3 $$\frac{\partial }{\partial \tau }{\Phi }_{{{b}}}=(-\frac{{\kappa }_{{{b}}}}{{\kappa }_{{{a}}}}-i{\alpha }_{{{b}}}-\Delta {k}_{{{b}}}\frac{\partial }{\partial \phi }+i{\beta }_{{{b}}}\frac{{\partial }^{2}}{\partial {\phi }^{2}}+i{\zeta }_{{{b}}}{\left|{\Phi }_{{{b}}}\right|}^{2}+i{\zeta }_{{{ab}}}{\left|{\Phi }_{{{a}}}\right|}^{2}){\Phi }_{{{b}}}+i{\mu }_{{{ab}}}{\Phi }_{{{a}}}^{2},$$

4 $$\frac{\partial }{\partial \tau }{\Phi }_{{{c}}}=(-\frac{{\kappa }_{{{c}}}}{{\kappa }_{{{a}}}}-i{\alpha }_{{{c}}}-\Delta {k}_{{{c}}}\frac{\partial }{\partial \phi }+i{\beta }_{{{c}}}\frac{{\partial }^{2}}{\partial {\phi }^{2}}+i{\zeta }_{{{c}}}{\left|{\Phi }_{{{c}}}\right|}^{2}+i{\zeta }_{{{ac}}}{\left|{\Phi }_{{{a}}}\right|}^{2}){\Phi }_{{{c}}}+i{\mu }_{{{ac}}}{\Phi }_{{{a}}}^{3},$$

where the electromagnetic field in the cavity is described by three slowly-varying field envelopes *Φ*_*a,b,c*_ for (a) infrared, (b) *χ*^(2)^, and (c) *χ*^(3)^ combs, respectively. The envelope *Φ*_*a*,*b*,*c*_ is normalized to ${\left|{\Phi }_{{a,b,c}}\right|}^{2}=2{g}_{{{a,b,c}}}{N}_{{{a,b,c}}}/{\kappa }_{{{a,b,c}}}$, where *N*_*a*,*b*,*c*_ is photon number in the corresponding fields. *κ*_*a*,*b*,*c*_ is the decay rate of the comb modes and ${g}_{{{a,b,c}}}={n}_{2}c\hslash {\omega }_{{{a,b,c}}}^{2}/({n}_{{{a,b,c}}}^{2}{V}_{{{a,b,c}}})$, where *ℏ* is the Planck’s constant, *c* is the speed of light in vacuum, *n*_2_ is the Kerr nonlinear index, *ω*_*a*,*b*,*c*_ is the resonance frequency at the center of each frequency band, *n*_*a*,*b*,*c*_ is the refractive index, and *V*_*a*,*b*,*c*_ is the effective mode volume. The envelopes are functions of *τ* and *ϕ*, where *τ* is the normalized time, *τ* = *κ*_a_*t*/2, *t* is the lab time, and *ϕ* is the cavity polar angle. *α*_*a*,*b*,*c*_ denotes normalized detuning and *f*_0_ is the normalized pump input. *β*_*a*,*b*,*c*_ denotes normalized second-order chromatic dispersion, and Δ*k*_*b*,*c*_ is the FSR mismatch between infrared combs and *χ*^(2)^ (*χ*^(3)^) combs in the unit of half cavity linewidth.

Parameters used in the LLE simulation are: *κ*_*a*_/2*π* = 1.21 × 10^7^ Hz, *κ*_*b*_/2*π* = 2.03 × 10^7^ Hz, *κ*_*c*_/2*π* = 3.05 × 10^7^ Hz, *β*_*a*_ = 1.8565, *β*_*b*_ = −4.1258, *β*_*c*_ = −6.0227, Δ*k*_*b*_ = −1.0179 × 10^3^, Δ*k*_*c*_ = −3.2310 × 10^3^, *ζ*_*b*_ = 1.6840, *ζ*_*c*_ = 2.5274, *ζ*_*ba*_ = 1.8738, *ζ*_*ca*_ = 1.1729, *ζ*_*ab*_ = 9.2780, *ζ*_*ac*_ =  13.0269, *μ*_*ba*_ = 0.0058, *μ*_*ca*_ = 0.2139, *μ*_*ab*_ = 0.0143, *μ*_*ac*_ = 0.7921, *f*_0_ = 26.1562. Parameters derived from experimental measurements are *κ*_*a*,*b*,*c*_, while other parameters are derived from 2D finite element method (FEM) simulation with rotation symmetry.

### 3D FDTD simulations of broadband coupling efficiency

The full 3D simulations of coupling efficiency are performed by a commercial simulator based on FDTD method. The microtoroid resonator is placed on the *x*–*y* plane. To make the simulation time affordable, the principal and minor diameters of the microresonator are scaled down to 24 and 4 μm, respectively. The thickness of the central disk is 2 μm. The nanofiber and the microfiber are represented by cylinders with diameters of 0.5 and 1.5 μm. A probe light source is applied to one side of the fiber. The excited mode in the fiber is set to be a fundamental mode. The material of the resonator is set to be silica with an additional imaginary part of 10^−5^ to its refractive index, and the material of the fiber is set to be silica without extra loss. Coupling efficiency is calculated as the difference of the base value and the dip value of the transmission spectrum. Different resonant modes are identified by field monitors inside the cavity. In Fig. 4b, a fundamental TE mode is used to calculate the coupling efficiency.

## Data Availability

Source data for Fig. 2 to Fig. 5 can be accessed at 10.6084/m9.figshare.12030408. Additional information is available from the corresponding authors upon reasonable request.

## Code availability

The codes that support the findings of this study are available from the corresponding authors upon reasonable request.
